# The Species Identification and Genomic Analysis of *Haemobacillus shengwangii*: A Novel Pathogenic Bacterium Isolated From a Critically Ill Patient With Bloodstream Infection

**DOI:** 10.3389/fmicb.2022.919169

**Published:** 2022-06-14

**Authors:** Yingying Du, Xuming Li, Yuhao Liu, Shikui Mu, Dandan Shen, Shu Fan, Zheng Lou, Shouqin Zhang, Han Xia, Yinghua Yuan, Sheng Wang

**Affiliations:** ^1^Department of Critical Care Medicine, School of Medicine, Shanghai Tenth People’s Hospital, Tongji University, Shanghai, China; ^2^Department of Scientific Affairs, Hugo Biotech Co., Ltd., Beijing, China; ^3^Department of Clinical Microbiology, School of Medicine, Shanghai Tenth People’s Hospital, Tongji University, Shanghai, China

**Keywords:** catheter-associated bloodstream infection, pathogenic bacterium, novel species identification, Thermicanaceae, genome *de novo* assembly, single-molecule real-time sequencing, comparative genomics

## Abstract

Since the first strain related to Thermicanaceae was reported in 1999, almost no literature on Thermicanaceae is available, particularly its genomics. We recently isolated a novel pathogenic bacterium, the ^△^ strain DYY3, from the blood sample of a critically ill patient. The morphological, physiological, and biochemical characteristics of ^△^ strain DYY3 were presented in this study, and the virulence factor genes and antibiotic resistance of DYY3 were also determined. Interestingly, the average nucleotide identity (ANI) and core-genes average amino acid identity (cAAI) analysis indicated that ^△^ strain DYY3 was genus novel and species novel. Moreover, phylogenetic analysis based on both 16S rRNA gene and whole genomic core gene sequences suggested that ^△^ strain DYY3 belonged to the family Thermicanaceae, and this novel taxon was thus named *Haemobacillus shengwangii* gen. nov., sp. nov. Besides, both the whole genome-based phylogenetic tree and amino acid identity analysis indicated that *Thermicanus aegyptius*, *Hydrogenibacillus schlegelii*, *Brockia lithotrophica*, and the newly discovered species *H. shengwangii* should belong to Thermicanaceae at the family level, and *T. aegyptius* was the closest species to *H. shengwangii*. We also constructed the first high-quality genome in the family Thermicanaceae using the next-generation sequencing (NGS) and single-molecule real-time (SMRT) sequencing technologies, which certainly contributed to further genomics studies and metagenomic-based pathogenic detection in the future.

## Introduction

Catheter-related bloodstream infection (CRBSI) is a frequent and life-threatening condition in the intensive care unit (ICU), which is associated with increased morbidity, mortality, and healthcare costs (Schwab et al., 2018; Gerver et al., 2020; Zeng et al., 2021). For example, according to a prospective multi-center study in China, the average incidence of CRBSI and the mortality due to CRBSI in ICU were 1.5/1,000 catheter days and 18.09%, respectively (Zeng et al., 2021). A similar incidence of CRBSI was reported in studies from European countries (Schwab et al., 2018; Gerver et al., 2020), and even higher rates were found in developing countries, up to 5.3/1,000 catheter days with 28–30% of mortality (Rosenthal et al., 2021).

To improve the clinical outcomes of patients with CRBSI, a rapid and accurate diagnosis of the causative pathogen is a critical step (Zhong et al., 2021). The current guideline recommends diagnosing CRBSI by hemoculture in suspected patients (Mermel et al., 2009), but this approach takes 48–96 h to isolate, identify, and perform antibiotic susceptibility tests (Tabak et al., 2018). Furthermore, it is often challenging to culture many fastidious or uncultivatable pathogens in standard automated systems (Murray and Masur, 2012). Therefore, emerging technologies, especially high-throughput sequencing, were attempted to replace conventional culture-based methods and initiate timely targeted anti-infection therapy (Li et al., 2021). However, few clinical studies thus far identified and classified unknown species with extraordinary genetic distances from known species.

The only reported bacteria that might belong to the family Thermicanaceae were *Thermicanus aegyptius*, *Hydrogenibacillus schlegelii*, *Brockia lithotrophica*, and *Carbobacillus altaicus*. *T. aegyptius* was first identified from soil and described as a fermentative microaerophile in 1999 (Gossner et al., 1999), and the reference genome was *T. aegyptius* DSM 12793, available online in 2013. *H. schlegelii* was originally named *Bacillus schlegelii* in 1979 (Schenk and Aragno, 1979) and was transferred to be a novel genus due to its massive divergence from other species in the genus *Bacillus* in 2013 (Kampfer et al., 2013). *H. schlegelii* was known for its ability of hydrogen-oxidizing (Barbosa et al., 2020) and was classified into order Bacillales and family Bacillaceae with NCBI taxonomy ID of 1484. *B. lithotrophica* was isolated from a hot spring in Russia and reported as a new taxon in 2013 (Perevalova et al., 2013). Finally, *C. altaicus* was still a candidate taxon classified into order Bacillales and family Bacillales *incertae sedis* with NCBI taxonomy ID 2163959. However, whether the classification method of the above bacteria is correct needs to be verified by genomic analysis, as this approach is increasingly being accepted as reliable data for bacterial taxonomy and species identification (Hayashi Sant’Anna et al., 2019).

In this study, the ^△^ strain DYY3, isolated from the blood sample of a critically ill patient diagnosed with CRBSI, cannot be identified by VITEK-MS automatic microbiological analyzer and the 16S rRNA sequence analysis. Thus, the high-quality genome of ^△^ strain DYY3 was constructed by the next-generation sequencing (NGS) and single-molecule real-time (SMRT) sequencing technologies, and multiple comparative genomics analyses were applied to identify this new strain.

## Materials and Methods

### Case Report

In January 2021, a 68-year-old female patient was admitted to the Shanghai Tenth People’s Hospital ICU due to acute respiratory failure, aspiration pneumonia, and cerebral infarction. Invasive mechanical ventilation, femoral vein catheterization, and urinary catheterization were performed during the treatment, and pulmonary infection was verified by fever, cough, and chest CT scanning. The patient’s infection was effectively controlled initially by the empirical use of ceftazidime. However, the patient’s body temperature, leukocyte count, and C-reactive protein were raised again after 9 days of anti-infection treatment. Considering the possibility of CRBSI, the femoral vein catheter was removed immediately, blood samples and the terminal of the central venous catheter were collected for bacterial culture, and vancomycin hydrochloride was added empirically to strengthen the anti-infection treatment. Three days later, both blood culture and catheter culture suggested unrecognized Gram-positive bacterial infection, and the drug sensitivity test showed that vancomycin was sensitive. Thus, vancomycin continued to be used, and the patient’s bloodstream infection was cured in 2 weeks. The strain designated DYY3 was isolated from the blood sample and preserved in a -80^°^C refrigerator to identify the unknown pathogenic bacterium further.

### Strain Isolation

The ^△^ strain DYY3 was isolated from a critically ill patient’s blood sample with a catheter-associated bloodstream infection. Briefly, the blood specimen was inoculated in a blood culture bottle (BD BACTEC Plus aerobic/F Culture Vials, Becton, Dickinson and Company, United States) at 35°C until it showed a positive result. For the isolation of ^△^ strain DYY3, blood agar plates (bioMérieux, Marcy l’Etoile, France) were used, and the plates were incubated in a CO_2_ incubator for 48 h. Later, dozens of single colonies were picked up from the blood agar plates. VITEK-MS automatic microbiological analyzer (bioMérieux, Marcy l’Etoile, France) was used to identify the taxonomic classification of the ^△^ strain DYY3 according to the standard operation process using the VITEK MS IVD KB V3.2 database as the reference. The total length of the 16S rRNA sequence was amplified by PCR using the primers of 27F (5′-AGAGTTTGATCMTGGCTCAG-3′) and 1492R (5′-GGTTACCTTGTTACGACTT-3′), and the amplified fragments were sequenced using a 3730XL sequencer (Applied Biosystems, United States).

### Morphology, Physiology, and Chemotaxonomy Analysis

Gram staining of ^△^ strain DYY3 was performed, referring to the procedures described by Wagner et al. (2018). The fresh biomass of DYY3 was stained with 1% (w/v) uranyl acetate, and the electron micrograph was taken by a transmission electron microscopy system JEM-1010 (JEOL, Japan). The growth tests were performed at various temperatures, NaCl concentrations, and pH levels using R2A agar plates (Difco, United States) as the culture medium. The tested temperatures were 4, 10, 15, 20, 30, 37, 40, 45, 50, and 55^°^C. The tested pH ranged from 5.0 to 11.0 with a gradient value of 1, K_2_HPO_4_/KH_2_PO_4_ buffer was used for pH 5–8, and NaHCO_3_/NaOH buffer was used for pH 9–11. The tested NaCl concentration ranged from 0 to 10% w/v using the interval of 1%. Acid production tests, enzyme activity tests, and additional phenotypic tests were performed using API 50CHB, API ZYM, and API 20NE galleries (bioMérieux, Marcy l’Etoile, France), respectively. The utilization of carbon sources was tested using Biolog GPIII Microplates (Biolog, United States), and quinones were extracted and identified using the HPLP LC-20AT system (Shimadzu, Japan). The fresh biomass of ^△^ strain DYY3 was hydrolyzed at 120°C for about 12 h to determine the composition of saccharides on its cell wall using ribose, arabinose, glucose, rhamnose, xylose, mannose, and galactose as references. The Cell Fatty Acid-Fatty Acid Methyl Ester (CFA-FAME) components were assayed by Agilent 6890 gas chromatograph (Agilent, United States), and the data were collected by the Sherlock Microbial Identification System (version 6.0, MIDI). The polar lipid analysis of ^△^ strain DYY3 (1 g of freeze-dried cells) was performed and examined by thin-layer chromatography (TCL) on cellulose sheets. The spots for polar lipids were identified by spraying with 10% phosphomolybdic acid in ethanol, a-naphthol, and ninhydrin, respectively.

### Antibiotic Susceptibility Test

The minimum inhibitory concentrations (MICs) of the ^△^ strain DYY3 to penicillin, ampicillin, vancomycin, gentamicin, erythromycin, ciprofloxacin, levofloxacin, clindamycin, trimethoprim/sulfisoxazole, rifampicin, and imipenem were determined by MicroScan Pos Combo Panel Type 33 (MicroScan, United States), with the interpretation of drug sensitivity results referred to the Clinical and Laboratory Standards Institute (CLSI) M45 guidelines.

### Genome Sequencing, Assembly, and Annotations

A 350-bp paired-end library was constructed and sequenced using the Illumina NovaSeq 6,000 sequencing platform (Illumina Inc., San Diego, CA, United States) with a PE150 layout. A 10-kb SMRT library was constructed and sequenced by the PacBio Sequel system (Pacific Biosciences, United States). The data were assembled by using Unicycler (version 0.4.7), and the genome was annotated by using Prokka (version 1.14.6) with default parameters. Prophages were annotated using phiSpy (version 4.2.12), and genomic islands were identified by using Islanpath-DIMOB (version 1.0.6). The BUSCO database (version 5.2.2) was used to evaluate the completeness of the genome sequence (Manni et al., 2021). BLASTp (version 2.10.1) was used to query the non-redundant (nr) protein sequence database and hit with the highest score, and the identity higher than 60 was recognized as a match for each gene. Eggnog-mapper (version 2.0.1) with the parameter of ‘‘seed_ortholog_evalue 1e-5-m diamond’’ was used to query the eggNOG database. HMMER (version 3.3.2) with the parameter of ‘‘-E 1e-5’’ was used for Pfam (version 33.1) database annotation, and Diamond (version 0.9.24.125) with the parameter of ‘‘-e 1e-5’’ was used for the Swiss-Port annotation. The genome atlas was plotted using CIRCOS.^1^ The virulence factor genes were predicted by querying VFDB using a web-based VFanalyzer. Antibiotic resistance genes were annotated by the CARD (version 3.1.4) database with BLASTp parameter of “-qcov_hsp_perc 80,” and hits with an identity less than 80 were filtered. The pathogen–host interaction associated genes were identified by the PHI database (version 4.12) with Diamond (version 0.9.24.125) parameter of “-e 1e-5” and hits with an identity less than 50 were filtered.

### Phylogenetic Relationship Analysis

The 16S rRNA maximum likelihood (ML) phylogenetic tree was constructed by RAxML^2^ using the GTR substitution matrix model. The whole genomic tree was constructed by IQ-TREE (version 1.6.12) with a bootstrap value of 1,000, referring to the core genes of the Genome Taxonomy Database (GTDB) (release202) (Rinke et al., 2021). One genome was selected as a representative for each genus of the principal families in class Bacilli. Two species from the family Acidaminococcaceae (phylum Firmicutes and class Negativicutes) and family Limnochordaceae (phylum Firmicutes and class Limnochordia) were set as out-group. The phylogenetic trees were plotted using iTOL.^3^ The average nucleotide identity (ANI) values and the values of the pairwise core-genes average amino acid identity (cAAI) were calculated using fastANI (Jain et al., 2018) and CompareM (version 0.1.2)^4^ with default parameters, respectively. Genes presented over 90% of genomes were used for cAAI calculation. The digital DNA-DNA hybridization (dDDH) values were calculated using a genome-to-genome distance calculator (GGDC)^5^.

## Results

### Morphology, Physiology, and Chemotaxonomy

**FIGURE 1:**
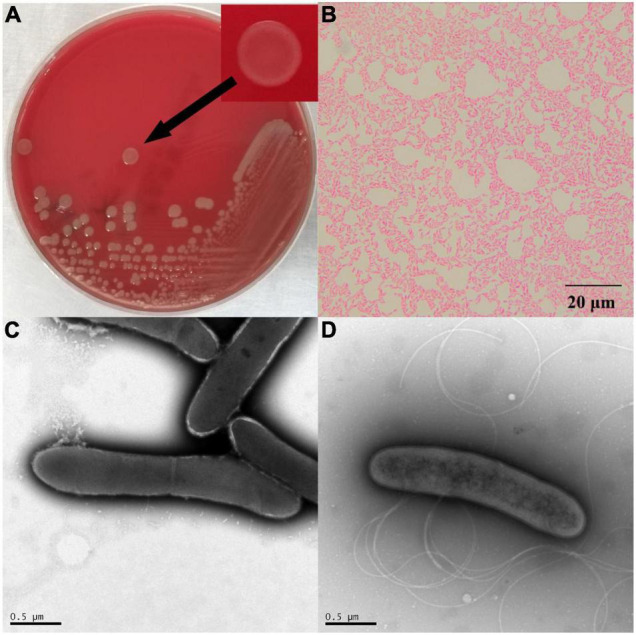
The morphology of strain DYY3. **(A)** The cultured bacterial colonial morphology on blood agar plate of ^△^ strain DYY3; **(B)** the Gram staining result shows that ^△^ strain DYY3 is Gram weak positive; **(C)** the electron microscope photograph of the dividing cells of ^△^ strain DYY3 after 24 h culture; **(D)** the electron microscope photograph shows the flagellum of ^△^ strain DYY3.

After 48 h of cultivation at 30°C, the colonies of ^△^ strain DYY3 on blood agar were 1–2 mm in size, grayish-white, round, smooth, and moist. The cells were weakly Gram-positive, rod-shaped, about 1.4–2.2 μm in length, and 0.4–0.5 μm in width, with flagellum and spore, motile, and facultative anaerobic (Figure 1). The ^△^ strain DYY3 grew well on R2A blood agar plates with a temperature range of 20–45^°^C (preferred 30–37^°^C). The strain can grow in NaCl concentration ranging from 0 to 2% w/v but not in NaCl concentration over 2%. In addition, the strain can also grow at a pH of 6–8 with an optimum of 7.

API galleries tests revealed inactive biochemical reactions of ^△^ strain DYY3 (Supplementary Table 1). The API ZYM assay indicated that alkaline phosphatase, esterase (C4), lipid esterase (C8), leucine aromatase, pancreatic rennet, acid phosphatase, and naphthol-AS-Bl-phosphate hydrolase tests were positive. The API 20NE assay suggested a positive assimilation test for glucose, arabinose, mannitol, mannose, N-acetyl glucosamine, maltose, gluconate, capric acid, adipic acid, malic acid, citric acid, and phenylacetic acid. However, the ^△^ strain DYY3 was only positive with 5-keto-gluconate in the API 50CHB assay.

Diphosphatidylglycerol (DPG), phosphatidylethanolamine (PE), and phosphatidylglycerol (PG) were identified to be the main polar lipids of ^△^ strain DYY3, and the two-dimensional TCL of the polar lipids photographs was shown in Supplementary Figure 1. The ^△^ strain DYY3 had no typical saccharides in the whole-cell hydrolysate experiment (Supplementary Figure 2). MK7 was the main methylanthraquinone in ^△^ strain DYY3. The fatty acid analysis revealed that DYY3 synthesized mainly iso- and anteiso-branched saturated fatty acids, mainly including C15:0 iso (61.75%), C15:0 anteiso (13.29%), C17:0 iso (3.56%), and C16:0 iso (3.31%), and a spot of unsaturated fatty acids C17:1 iso w10c (3.54%). According to the RTSBA6 (version 6.21) database, *Bacillus* was the closest genus, but the similarities were not high (26.70%; refer to Supplementary Tables 2A,B). Carbon source utilization assays showed that ^△^ strain DYY3 only used D-serine and glucuronamide as carbon sources (Supplementary Table 3). The VITEK-MS typing results cannot assign ^△^ strain DYY3 to any species in the database, but later genetic analysis (see below) suggested that DYY3 belonged to the family Thermicanaceae. Hence, the morphological, physiological, and biochemical characteristics of the four species in the family Thermicanaceae are summarized in Table 1.

**TABLE 1:** Characteristics of *Haemobacillus shengwangii* and close species.

Characteristics	*Haemobacillus shengwangii* DYY3	*Thermicanus aegyptius* DSMZ 12793^T^	*Brockia lithotrophica* Kam1851^T^	*Hydrogenibacillus schlegelii* DSM 2000^T^
Cell shape	Rod	Rod	Rod	Rod
Gram reaction	Week positive	Week positive	Positive	Positive
Motility	+	+	+	+
Spores	+	-	+	+
Flagellum	+	+	+	+
Colony color	Grayish white	Geige	NA*	Cream
Temperature for growth				
Range	20–45^°^C	37–65^°^C	46–78^°^C	37–80^°^C
Optimum	30–37^°^C	55–60^°^C	60–65^°^C	70–75^°^C
pH for growth				
Range	6–8	5.5–7.7	5.5–8.5	4.2–7.5
Optimum	7	6.5–7	6.5	6–7
NaCl concentration for growth (%, w/v)				
Range	0–2	NA	NA	3–5
Optimum	0	NA	NA	3
Oxygen requirement	Facultative anaerobic	Facultative anaerobic	Strictly aerobic	Strictly aerobic
DNA G + C content (mol%)	40.62	50.3	63	67–68
Major fatty acids	C15:0 iso, C15:0 anteiso, C17:0 iso, C16:0 iso	NA	C16:0, C16:iso, C18:0, C17:0	C16:0 iso
Polar lipids^ #^	DPG, PG, PE	NA	NA	DPG, PG
Quinone	MK7	NA	NA	MK7

*^#^DPG, diphosphatidylglycerol; PG, phosphatidylglycerol; PE, phosphatidylethanolamine.*

*NA, not available.

### 16S rRNA Phylogenetic Tree

**FIGURE 2:**
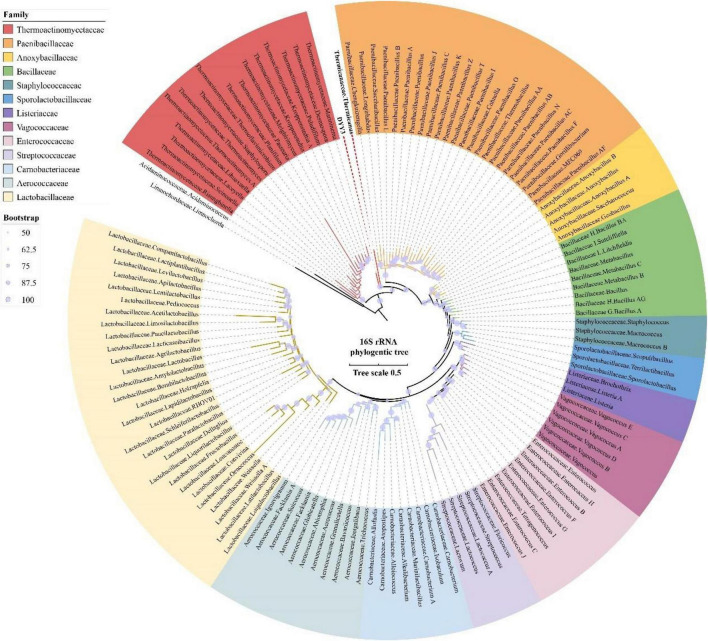
16S rRNA gene tree of strain DYY3. The rooted 16S rRNA gene ML tree was constructed by RAxML utilizing the typical species of Bacilli and also the matched species on NCBI with a bootstrap value of 1,000. Acidaminococcaceae, Acidaminococcus, Limnochordaceae, and Limnochorda were set as the out-group, and the other species were Bacilli in class.

To identify the phylogenetic relationship of ^△^ strain DYY3, we first amplified and sequenced its full-length region of 16S rRNA genes. Then, we queried the 16S rRNA sequence at NCBI using online BLASTn. The result showed that the taxon of ^△^ strain DYY3 was close to *Bacillus* at the genus level, and belonged to *Bacillus* at class level, and the best matching degree was 94.49%, and all the hit taxon belonged to Bacilli in class. Later, we extracted the entire length of the 16S rRNA gene sequence to construct a phylogenetic tree, utilizing the typical Bacilli species and the matched species on NCBI (Figure 2). The sequence information, blast results, and sequence similarity are shown in Supplementary Table 4. The phylogenetic tree showed that ^△^ strain DYY3 was close to *Thermicanus* at the genus level and might belong to Thermicanaceae at the family level. However, this new taxon had six copies of the 16S rRNA gene, which were 100% identical. Therefore, contradictory conclusions were drawn by the online NCBI blast results and sequence information of the 16S rRNA phylogenetic tree; thus, ^△^ strain DYY3 cannot be assigned to any existing species or genus.

### Genome Features

**FIGURE 3:**
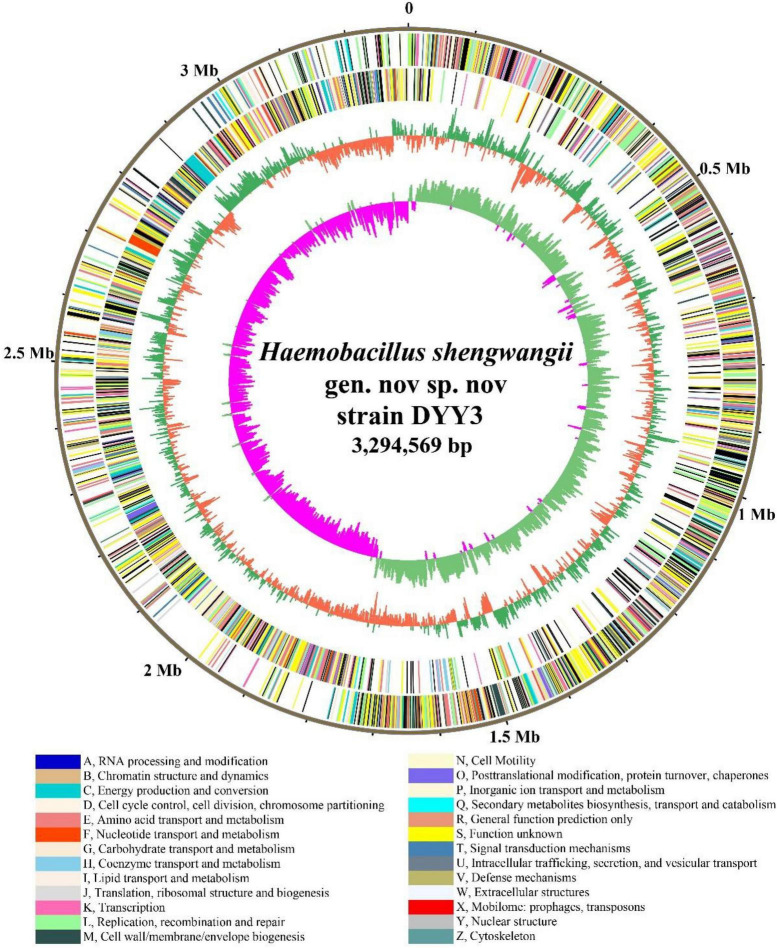
Genome atlas of strain DYY3. The outer black circle shows the genome coordinates, and the next two circles represent forward and reverse strand CDSs with colors representing the functional classification of COG. The last two circles are GC content and GC skew using a 5-kb window overlapping at 1,000 bp. The COG functional classifications and colors are shown at the bottom of the figure. The green and orange colors of the fourth circle mean the GC content is higher and lower than the average GC content of the genome, respectively. The inner circle’s purple and light blue colors show the GC-skew values lower and higher than 0, respectively.

We combined NGS and SMRT technologies to construct the genome of ^△^ strain DYY3 (Supplementary Tables 5A,B), and a circular genome with a total length of 3,294,569 bp and Guanine and Cytosine content of 40.62% was obtained (Figure 3 and Table 2). All conserved BUSCO genes (100%) were identified within the genome (Supplementary Table 6), indicating the high quality of this constructed genome. A total of 3,264 CDSs with an average length of 861 bp, 18 rRNAs, and 72 tRNAs were predicted. Besides, thirteen genomic islands (Supplementary Table 7) and two partial prophage regions (Supplementary Table 8) were identified in the genome. A total of 3,003 (89.72%) coding seuqence could be assigned with annotations, and 366 (10.94%) CDSs, including 22 database-based annotations, were annotated as hypothetical proteins (Table 3 and Supplementary Tables 9A–D).

**TABLE 2:** Genome features of strain DYY3.

Genome features	Values
Chromosome	1 (circular)
Genome size	3,294,569
Genome coverage (NGS)	577
Genome coverage (TGS)	256
G + C content (%)	40.62%
BUSCO	100%
rRNAs (5, 16, 23S)	18 (6, 6, 6)
tRNAs	72 (35 families)
CDS genes	3,347
Genomic Island	13

**TABLE 3:** Gene functional annotation of strain DYY3.

Database	Annotated gene number	Percentage
Nr	1,834	54.80%
Pfam	2,685	80.22%
Swiss-Prot	2,034	60.77%
EggNOG	2,935	87.69%
GO	697	20.82%
KEGG	1,990	59.46%
COG	2,741	81.89%
Total	3,003	89.72%

### Phylogenetic Relationship

**FIGURE 4:**
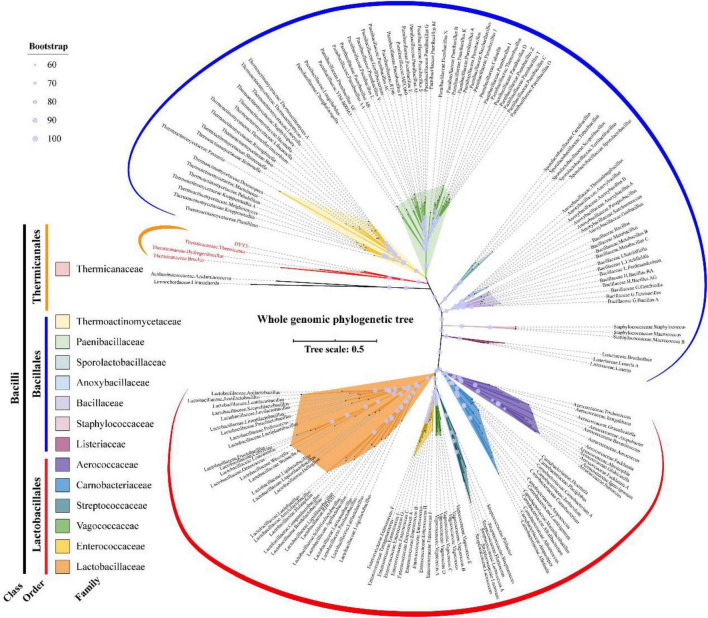
Whole genome-based phylogenetic relationship of strain DYY3. The unrooted whole genomic maximum likelihood tree was constructed by IQ-TREE using GTDB-based 120 conserved core genes with a bootstrap value of 1,000. Acidaminococcaceae, Acidaminococcus, Limnochordaceae, and Limnochorda were set as the out-group, and the other species were Bacilli in class. The 14 families of the Bacilli class were differently colored, and the bootstrap values for each node were shown by the size of a light blue circle.

**FIGURE 5:**
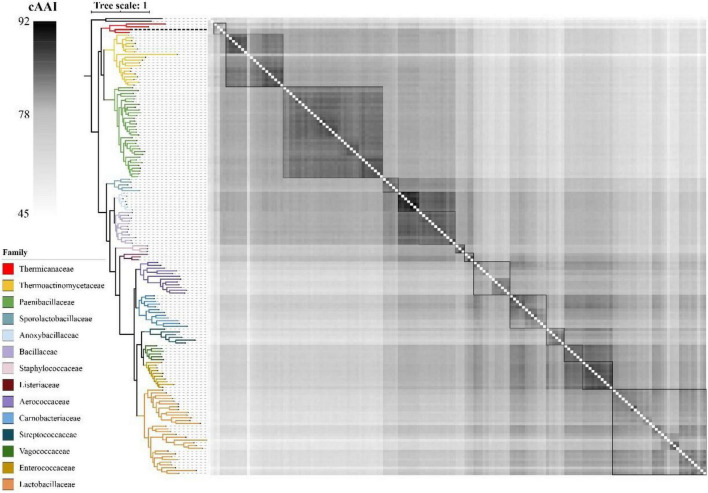
Heatmap depicting the cAAI values of the 165 pairwise comparisons in class Bacilli. The left phylogenetic tree shows the phylogenetic relationships of different families, and the right heatmap shows the cross matrix of the core-genes average amino acid identity values between each genus. The order of each branch is identical to that of Figure 4. The black dash line shows ^△^strain DYY3.

As shown in Figure 4, the whole genome-based phylogenetic tree was constructed, employing the protein sequence of the 120 conserved genes from the GTDB database, and 165 strains, including all the possible and available genomes of the Thermicanaceae family, were selected. The genomes’ source, information, and dDDH are listed in Supplementary Table 10. This whole genome-based phylogenetic tree showed high accordance with the 16S rRNA phylogenetic tree, demonstrating that DYY3 was close to the genus *Thermicanus* and belonged to the same taxon at the family level. Moreover, the sequences of majority groups were significantly distinct from calculating reliable values as the collinear regions were mainly less than 1% of the whole genomes. We then calculated cAAI to show the divergence between each genus, considering that the amino acid sequence is more conserved than the nucleotide sequence of gene coding regions. The plotted heatmap of the cAAI values agreed well with the core genes-based phylogenetic tree (Figure 5 and Supplementary Table 11), suggesting that ^△^ strain DYY3 belonged to Thermicanaceae at the family level. The average cAAI value between DYY3 and species from other families was 55.68, and the average cAAI value of each genus within the same existing family was 70.22 (Table 4). The cAAI value between *T. aegyptius* and DYY3 was 67.21, close to the average cAAI value for a single family, further demonstrating that ^△^ strain DYY3 belonged to Thermicanaceae at the family level.

**TABLE 4:** Statistical result of cAAI values between different families in Bacilli class.

Family name	Average cAAI values between DYY3 and each family	Average cAAI values between each genus in a single family	Genomes in each family
Thermoactinomycetaceae	59.59	68.25	19
Paenibacillaceae	60.47	70.38	33
Sporolactobacillaceae	58.73	70.23	5
Anoxybacillaceae	60.67	83.38	7
Bacillaceae	59.76	72.48	4
Staphylococcaceae	54.64	73.17	3
Listeriaceae	56.11	70.51	3
Aerococcaceae	51.74	61.95	12
Carnobacteriaceae	53.04	66.4	12
Streptococcaceae	50.91	66.75	6
Vagococcaceae	53.64	72.11	6
Enterococcaceae	53.65	75.34	10
Lactobacillaceae	50.91	61.86	31
**Average**	**55.68**	**70.22**	**12**
**Min**	**50.91**	**61.86**	**3**
**Max**	**60.67**	**83.38**	**33**
***T. aegyptius* vs. strain DYY3**	**67.21**		

Finally, we compared the whole genomic similarity within the possible and available genomes of the Thermicanaceae family using BLAST-based ANI (ANIb) (Table 5). The ANIb between DYY3 and *T. aegyptius* was calculated to be 69.51%, indicating that they did not belong to the same species, as generally 95% ANI was found to recapitulate the majority species (Jain et al., 2018; Olm et al., 2020; Parks et al., 2020). More importantly, *T. aegyptius*, *B. lithotrophica*, and *H. schlegelii* represented three different genera, and the ANIbs within the four species (e.g., DYY3) ranged from 68.86 to 75.40%, suggesting that ^△^ strain DYY3 did not belong to any existing genus. Therefore, ^△^ strain DYY3, the novel taxon, was named *Haemobacillus shengwangii* gen. nov., sp. nov. *Haemobacillus* referred to the strain isolated from the blood and was spore-forming and rod-shaped, and *Shengwangii* was named for appreciating the outstanding effort of Doctor Wang to save patients’ lives.

**TABLE 5:** The ANIb values and alignment length of strain DYY3 and species in family Thermicanaceae.

Species		Alignment length			
		*H. shengwangii* DYY3	*T. aegyptius* DSM 12793	*B. lithotrophica a* DSM 22653	*H. schlegelii* MA48
**ANIb**	**DYY3**		304,571	36,373	65,834
	***T. aegyptius***	69.51%		77,647	176,981
	***B. lithotrophica***	69.27%	68.86%		301,196
	***H. schlegelii***	68.82%	69.75%	75.40%	

### Virulence Factors

A total of 30 genes of 16 virulence factor classes were predicted to encode putative virulence factors in ^△^ strain DYY3′s genome by the virulence factor database (VFDB). DYY3 owned eight unique predicted virulence factor genes associated with adherence, anti-phagocytosis, immune evasion, intracellular survival, iron uptake, and motility (Supplementary Table 12). In addition, twenty genes were predicted associated with hypervirulence in DYY3′s genome using the Pathogen Host Interactions database (PHI-base), with the highest number of the four species in the Thermicanaceae family (Supplementary Tables 13A,B).

### Antibiotic Resistance

Two genes, shared by ^△^ strain DYY3 and *T. aegyptius* DSMZ 12793T, were predicted to be associated with antibiotic resistance (Supplementary Table 14). Of the two genes, one gene was related to ARO:3003438, annotated as AMR Gene Family associated with elfamycin. Another gene was related to ARO:3002838, annotated as LNU lincosamide nucleotidyltransferase associated with lincosamide. Besides, the MIC test revealed that ^△^ strain DYY3 was sensitive to the most commonly used antibiotics, including penicillin, ampicillin, vancomycin, gentamicin, erythromycin, ciprofloxacin, levofloxacin, clindamycin, rifampicin, imipenem, and trimethoprim/sulfamethoxazole (Supplementary Table 15).

## Discussion

Traditional morphological, physiological, and biochemical studies and comparative genomic analysis demonstrated that the ^△^ strain DYY3 was a novel bacterial pathogen belonging to class Bacilli, order Thermicanales, family Thermicanaceae, and genus *Haemobacillus*. We thus proposed *H. shengwangii* gen. nov., sp. nov. to be the name of the novel taxon. In addition, our data supported that *T. aegyptius*, *H. schlegelii*, *B. lithotrophica*, and *H. shengwangii* should belong to Thermicanaceae at a family level. Among the genome published in the family Thermicanaceae, *H. shengwangii* was the first genome constructed with high quality, which undoubtedly contributed to the future research of this family.

Based on 16S rRNA gene sequencing, querying, and phylogenetic tree analysis, ^△^ strain DYY3 was identified as a species belonging to class Bacilli, and *T. aegyptius* was the most adjacent taxon. However, when we investigated the taxonomy background of the genus *Thermicanus*, the taxonomic status of this genus was inconsistent at the family level in different authoritative taxonomic databases, including the NCBI taxonomy database, GTDB, SILVA, and GBIF database. We then constructed the core genes-based phylogenetic tree using 165 representative species, including the typical genus of the representative families in class Bacilli and the genus associated with the family Thermicanaceae. The phylogenetic tree indicated that *T. aegyptius*, *H. schlegelii*, *B. lithotrophica*, and ^△^ strain DYY3 were in the same tree clade, and the cAAI heatmap further verified their close genetic relationships. Hence, these four species should be classified into the same family. *T. aegyptius* was the closest species to ^△^ strain DYY3, supported by the 16S rRNA gene tree and the whole genomic phylogenetic tree. The calculated ANIb (69.51%) between ^△^ strain DYY3 and *T. aegyptius* str. DSM 12793 was far from the standard (95%) belonging to the same species, which indicated that ^△^ strain DYY3 was a novel species. Since three of the four species in the family Thermicanaceae were known as different genera, the narrow range of the ANIb values (68.86–75.40%) suggested that DYY3 also did not belong to any existing genus. Therefore, ^△^ strain DYY3 was verified belonging to a genus novel and species novel taxon in Thermicanaceae. This new taxon was proposed to be named *Haemobacillus shengwangii* gen. nov., sp. nov.

The MIC test showed that ^△^ strain DYY3 was sensitive to all commonly used antibiotics. Ceftazidime was initially used in the patient’s antibiotic treatment, which turned ineffective. Subsequently, vancomycin was empirically added to the anti-infection therapy, and the symptoms of infection were improved rapidly. Since *H. shengwangii* is a Gram-positive, penicillins, glycopeptides, and cephalosporins should be recommended. In addition, the MIC test also indicated that ^△^ strain DYY3 did not have antibiotic resistance, although the CARD database predicted two antibiotic resistance genes associated with elfamycin and lincosamide, respectively. To further clarify the virulence of ^△^ strain DYY3, its virulence factor genes were predicted based on sequence similarity comparison against the VFDB. The ^△^ strain DYY3 owned eight unique predicted virulence factor genes associated with adherence, anti-phagocytosis, immune evasion, intracellular survival, iron uptake, and motility. Besides, twenty genes were predicted to be associated with hypervirulence in the ^△^ strain DYY3 genome using PHI-base, with the highest number of the four species in the Thermicanaceae family. Given that *Bacillus* was the closest genus that can be queried in the database, the phylogenetic relationships of ^△^ strain DYY3 and species in genus *Bacillus* were quite distant, and the sequence similarities of ^△^ strain DYY3 and species in genus *Bacillus* were relatively low; we cannot exclude the possibility of ^△^ strain DYY3 owning more genes associated with virulence factors.

Although blood culture is the golden standard for pathogenic diagnosis, it cannot fully support that ^△^ strain DYY3 was the only pathogenetic bacterium responsible for this patient’s infection, as only a tiny proportion of pathogens are identifiable by the culture-based methods (Li et al., 2021). mNGS was more suitable for pathogenic detection when the pathogen was unculturable, novel, or variant species, such as DYY3, as all the nucleic acids can be sequenced and analyzed indiscriminately (Wilson et al., 2019; Price et al., 2021), and NGS has been widely used to perform comprehensive and precise diagnosis of pathogens with various sample types (Miller et al., 2019; Chen et al., 2021; Gu et al., 2021). Nonetheless, without a reference genome, even if the genome of a pathogen was sequenced, the species information cannot be disclosed by mNGS. Therefore, the availability of the *H. shengwangii* genome sequence could provide a valuable source for further comparative genomics analysis in the family Thermicanaceae and facilitate the family’s detection rate when conducting an mNGS-based pathogenic detection or study.

According to the conventional taxonomic features, *T. aegyptius*, *H. schlegelii*, *B. lithotrophica*, and *H. shengwangii* shared the identical phenotypes of rod-shaped, Gram-positive, spore-forming, and motor ability, indicating that these were the standard features of family Thermicanaceae. However, a clear description of the Thermicanaceae needs further studies, as few reference species and literature on Thermicanaceae are currently available. In addition, we did not trace the source of ^△^ strain DYY3, such as the patient’s skin, living environment, and so on, and only one strain of this novel species was isolated so far. Finally, the pathogenic mechanism was not thoroughly investigated, although virulence factor genes and antibiotic resistance genes were predicted in this study.

## Conclusion

This study identified a novel pathogenic bacterium, *H. shengwangii* gen. nov., sp. nov., isolated from a critically ill patient with CRBSI. In addition to the traditional methods of species identification, we used multiple comparative genomics analyses to prove that ^△^ strain DYY3 was genus novel and species novel in the family of Thermicanaceae. Moreover, the constructed high-quality *H. shengwangii* genome will contribute to further genomics research and NGS-based pathogenic detection or study in the family Thermicanaceae.

## Data Availability Statement

The datasets presented in this study can be found in online repositories. The names of the repository/repositories and accession number(s) can be found in the article/Supplementary Material.

## Ethics Statement

Written informed consent was obtained from the individual(s) for the publication of any potentially identifiable images or data included in this article.

## Author Contributions

SW and YY conceived and designed the project. YD, YL, DS, and SM participated in the strains collection. YD, YL, and SZ performed the morphology physiology and chemotaxonomy identification test. XL, SF, and HX conducted the bioinformatic analysis. YD and XL wrote the manuscript. SW and ZL revised the manuscript. All authors contributed to the article and approved the submitted version.

## Conflict of Interest

XL, SF, HX, and ZL were employed by Hugo Biotechnologies Co., Ltd. The remaining authors declare that the research was conducted in the absence of any commercial or financial relationships that could be construed as a potential conflict of interest.

## Publisher’s Note

All claims expressed in this article are solely those of the authors and do not necessarily represent those of their affiliated organizations, or those of the publisher, the editors and the reviewers. Any product that may be evaluated in this article, or claim that may be made by its manufacturer, is not guaranteed or endorsed by the publisher.

## Footnotes

1
http://circos.ca/

2
https://raxml-ng.vital-it.ch/#/

3
https://itol.embl.de/

4
http://github.com/dparks1134/CompareM

5
http://ggdc.dsmz.de/

## Supplementary Material

Supplementary Figure 1 Two-dimensional TLC of polar lipids photographs from strain DYY3. Figure **(A–E)** shows the dyeing results using ninhydrin, molybdenum blue 1-methylnaphthol, d reagent, and molybdophosphate. DPG, diphosphatidylglycerol; PG, phosphatidylglycerol; PE, phosphatidylethanolamine; PL1–5, unidentified phospholipids; GL, unidentified glycolipid; L1–2, unidentified lipids; AGPL, unidentified amino-glycerophospholipid.

Supplementary Figure 2 Whole-cell saccharide hydrolysate experiment. The results indicated that ^△^ strain DYY3 did not have any characteristic sugars.
