# Supplementary figures and images for: The Species Identification and Genomic Analysis of Haemobacillus shengwangii: A Novel Pathogenic Bacterium Isolated From a Critically Ill Patient With Bloodstream Infection
Source: Front Microbiol. 2022 Jun 14;13:919169. doi: 10.3389/fmicb.2022.919169 (PMC9237643; doi:10.3389/fmicb.2022.919169)

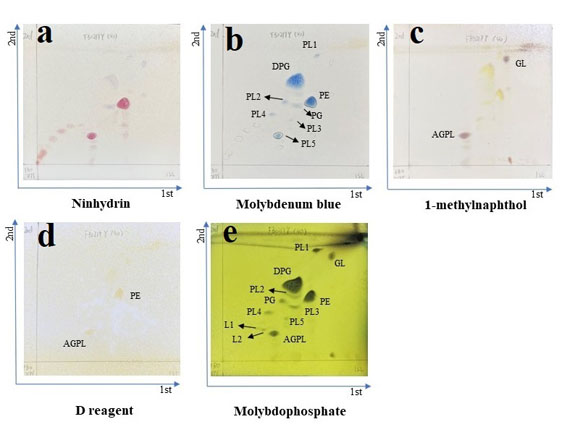

Supplement: Supplementary Figure 1 — Two-dimensional TLC of polar lipids photographs from strain DYY3. Figure (A–E) shows the dyeing results using ninhydrin, molybdenum blue 1-methylnaphthol, d reagent, and molybdophosphate. DPG, diphosphatidylglycerol; PG, phosphatidylglycerol; PE, phosphatidylethanolamine; PL1–5, unidentified phospholipids; GL, unidentified glycolipid; L1–2, unidentified lipids; AGPL, unidentified amino-glycerophospholipid. [file Image_1.JPEG]

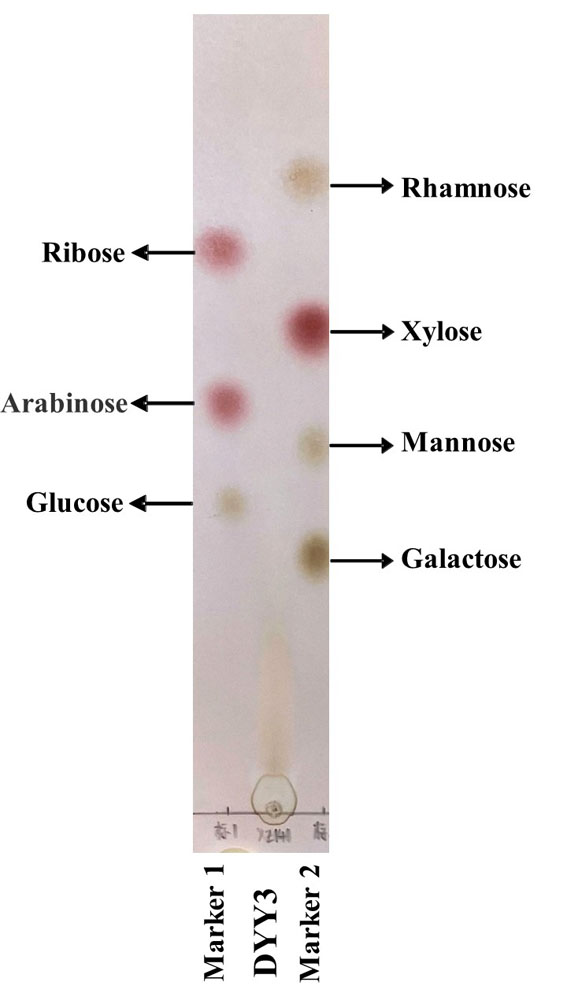

Supplement: Supplementary Figure 2 — Whole-cell saccharide hydrolysate experiment. The results indicated that △ strain DYY3 did not have any characteristic sugars. [file Image_2.JPEG]
